# Does genetic heterogeneity account for the divergent risk of type 2 diabetes in South Asian and white European populations?

**DOI:** 10.1007/s00125-014-3354-1

**Published:** 2014-08-22

**Authors:** Zahra N. Sohani, Wei Q. Deng, Guillaume Pare, David Meyre, Hertzel C. Gerstein, Sonia S. Anand

**Affiliations:** 1 Population Genomics Program, Department of Clinical Epidemiology and Biostatistics, McMaster University, 1280 Main St W, Hamilton, ON Canada L8S 4L8; 2 Chanchlani Research Centre, McMaster University, Hamilton, ON Canada; 3 Population Health Research Institute, Hamilton Health Sciences, Hamilton, ON Canada; 4 Department of Pathology and Molecular Medicine, McMaster University, Hamilton, ON Canada; 5 Department of Medicine, McMaster University, Hamilton, ON Canada

**Keywords:** Epidemiology, Ethnicity, Genetic risk, Meta-analysis, South Asian, Type 2 diabetes, White Europeans

## Abstract

**Aims/hypothesis:**

South Asians are up to four times more likely to develop type 2 diabetes than white Europeans. It is postulated that the higher prevalence results from greater genetic risk. To evaluate this hypothesis, we: (1) systematically reviewed the literature for single nucleotide polymorphisms (SNPs) predisposing to type 2 diabetes in South Asians; (2) compared risk estimates, risk alleles and risk allele frequencies of predisposing SNPs between South Asians and white Europeans; and (3) tested the association of novel SNPs discovered from South Asians in white Europeans.

**Methods:**

MEDLINE, Embase, the Cumulative Index to Nursing and Allied Health Literature (CINAHL) and the Cochrane registry were searched for studies of genetic variants associated with type 2 diabetes in South Asians. Meta-analysis estimates for common and novel bi-allelic SNPs in South Asians were compared with white Europeans from the DIAbetes Genetics Replication And Meta-analysis (DIAGRAM) consortium. The population burden from predisposing SNPs was assessed using a genotype score.

**Results:**

Twenty-four SNPs from 21 loci were associated with type 2 diabetes in South Asians after meta-analysis. The majority of SNPs increase odds of the disorder by 15–35% per risk allele. No substantial differences appear to exist in risk estimates between South Asians and white Europeans from SNPs common to both groups, and the population burden also does not differ. Eight of the 24 are novel SNPs discovered from South Asian genome-wide association studies, some of which show nominal associations with type 2 diabetes in white Europeans.

**Conclusions/interpretation:**

Based on current literature there is no strong evidence to indicate that South Asians possess a greater genetic risk of type 2 diabetes than white Europeans.

**Electronic supplementary material:**

The online version of this article (doi:10.1007/s00125-014-3354-1) contains peer-reviewed but unedited supplementary material, which is available to authorised users.

## Introduction

Type 2 diabetes is a metabolic disorder resulting from the interplay between insulin secretion and action. The prevalence of type 2 diabetes is increasing globally [1]. Currently, there are 61 million people with diabetes in the Indian subcontinent, a number that will rise to 100 million by the year 2030, representing 25% of the world’s burden [2, 3]. South Asians develop metabolic abnormalities, such as hyperglycaemia, low HDL-cholesterol, and elevated triacylglycerol, at a lower BMI and younger age than white Europeans [4–6]. It is hypothesised that the higher prevalence of type 2 diabetes results from a greater genetic predisposition among South Asians [7, 8], either because of larger risk estimates for each single nucleotide polymorphism (SNP) or increased frequency of risk alleles. This hypothesis has not been systematically evaluated.

The purpose of this systematic review is to: (1) establish risk estimates for SNPs predisposing South Asians to type 2 diabetes; (2) compare risk estimates, risk alleles and risk allele frequencies (RAFs) of type 2 diabetes predisposing SNPs between South Asians and white Europeans; and (3) explore the association of novel SNPs discovered from South Asians in a large cohort of white Europeans.

## Methods

### Systematic review of studies assessing genetic risk of type 2 diabetes in South Asians

#### Search strategy and selection criteria

MEDLINE, Embase, the Cumulative Index to Nursing and Allied Health Literature (CINAHL) and the Cochrane registry (from inception to 17 June 2013; MEDLINE and Embase searched using OvidSP) were searched for studies of genetic variants associated with type 2 diabetes in South Asians. South Asians were defined as individuals originating from India, Pakistan, Bangladesh or Sri Lanka. The search strategy was developed in consultation with a research librarian and did not restrict by type of genetic variant, language of study or study design (i.e. case–control, cohort, cross-sectional). The full search strategy is presented in electronic supplementary material (ESM) Table 1. Experts were consulted, and reference lists of included articles as well as relevant excluded articles were searched. Two reviewers (ZNS and WQD) independently assessed each study for eligibility based on four questions: (1) is at least one study population South Asian; (2) is type 2 diabetes the outcome studied; (3) is the exposure a genetic variant; and (4) is this a genetic association study? Disagreements were independently resolved by a third reviewer (SSA). Articles that passed the screening phase were reviewed in depth.

#### Full-text review and data extraction

Conference abstracts, narrative reviews and other systematic reviews were excluded after full-text review. Primary studies investigating genetic variants other than bi-allelic SNPs (e.g. insertions, deletions, length polymorphisms, haplotypes and complex tri-allelic SNPs) or with fewer than 25 cases were excluded. These restrictions were implemented to ensure that included studies could be combined and were of sound quality. Additionally, to ensure robustness of our results, SNPs from included studies carried forward to meta-analysis were limited to those that previously reached genome-wide significance in any ethnicity. If datasets were published more than once, publication with the largest sample size or the one recommended by the senior author was used for meta-analysis. Two reviewers conducted a full-text assessment and extracted data on participant characteristics (sample size, age, BMI, fasting glucose, waist and hip circumferences and % men), study design (study methods, region) and results (SNP, risk allele, RAF in cases and controls, and risk estimate with a 95% CI). For genome-wide association studies (GWAS) in South Asians, SNPs associated with type 2 diabetes at *p* < 10^−3^ were considered for meta-analysis.

#### Quality assessment of included studies

Using recommendations from the STrengthening the REporting of Genetic Association Studies (STREGA) guidelines [9], the following three components were considered for quality assessment: testing for Hardy–Weinberg equilibrium (HWE) in controls [10], reporting of genotyping call rate or appraisal of genotyping quality by duplication, and sources of case and control ascertainment.

#### Statistical analysis

Agreement between reviewers was reported with Cohen’s *κ* statistic. Allelic ORs were calculated using RAFs and sample size for cases and controls and assessed for significance using Fisher’s exact test. The ORs were not adjusted for any covariates. ORs for SNPs in linkage disequilibrium (LD) (*r* ^2^ > 0.8) obtained from independent cohorts were meta-analysed using a random-effects model weighted by inverse variance. LD was estimated using 1000 Genome Centre d'Etude du Polymorphisme (CEU) (Utah residents with northern and western European ancestry) data from SNAP (Broad Institute, Cambridge, MA, USA) [11]. A two-sided *α* level of 0.05 was considered significant. For a list of SNPs in LD, see ESM Table 2. Summary RAFs are presented as averages, weighted by sample size, of all included reports. Heterogeneity was estimated using *I* ^2^ statistic, which indicates the proportion of total variation in estimates attributed to heterogeneity, as well as the *Q* statistic [12]. A cut-off of 25% for *I* ^2^ was used to represent minimal heterogeneity, 50% to represent moderate and 75% to represent high heterogeneity. Quanto, Los Angeles, CA, USA (version 1.2.4) was used for sample size and power calculations assuming a disease prevalence of 10% and an additive model of inheritance. All other statistical analyses were conducted in R (version 3.0.2).

### Comparison of meta-analysed SNPs in South Asians with white Europeans

Estimates for SNPs associated with type 2 diabetes in this meta-analysis were retrieved for white Europeans from the DIAbetes Genetics Replication And Meta-analysis (DIAGRAM) consortium. Specific details on the consortium have been previously described [13]. Briefly, DIAGRAM stage 1 is a publicly available database of 12 GWAS with 12,171 cases and 56,862 controls. DIAGRAM authors tested SNPs with minor allele frequency >1% for association with type 2 diabetes under an additive model. The authors combined summary estimates from the 12 GWAS using fixed-effect inverse-variance-weighted meta-analysis.

Effect sizes from white Europeans in DIAGRAM and South Asians from this meta-analysis were transformed to natural logs and compared using a *Z* test. RAFs in white Europeans were acquired from published GWAS (www.genome.gov/gwastudies/). Additionally, a genotype score of SNPs present in both groups was constructed to compare population burden from these SNPs. The genotype score was constructed as below:

$$ \mathrm{Genotype}\ \mathrm{score}=\Sigma\ \left[{ \log}_e\left({\mathrm{OR}}_{\mathrm{i}}\right)\times {\mathrm{RAF}}_{\mathrm{i}}\right] $$

Variance for the genotype score was estimated as:

$$ \mathrm{Variance}=\Sigma \left\{{{\mathrm{RAF}}_{\mathrm{i}}}^2\times \mathrm{variance}\left[{ \log}_e\left({\mathrm{OR}}_{\mathrm{i}}\right)\right]\right\} $$

Genotype scores from both ethnicities were compared using a *Z* test.

### Testing novel SNPs discovered from South Asians GWAS in white Europeans

**Fig. 1:**
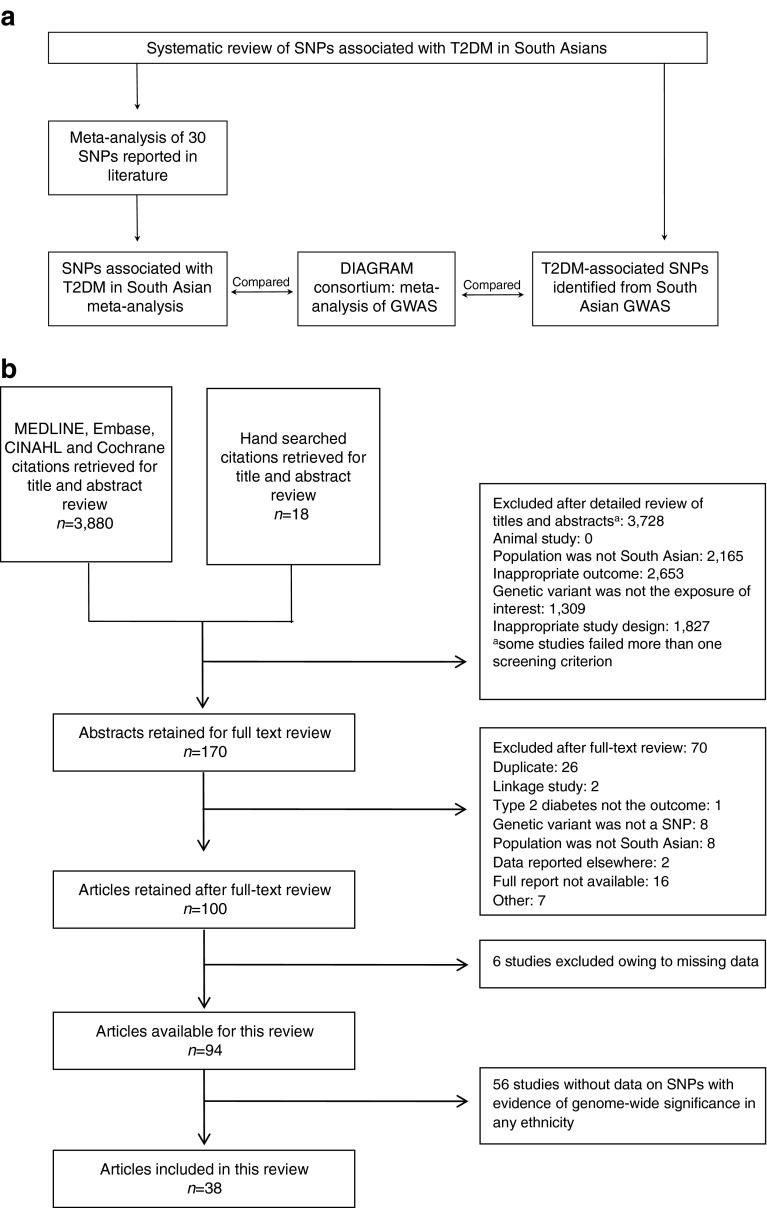
(**a**) Overview of the study design. (**b**) Flow diagram of the systematic review of South Asian literature investigating genetic variants predisposing to type 2 diabetes (first objective of study). T2DM, type 2 diabetes mellitus

Four GWAS have been conducted in South Asians [14–17], which collectively identified nine SNPs (*p* < 5 × 10^−8^). All studies used a case–control design. Associations with type 2 diabetes for eight of the nine SNPs have not been independently discovered from GWAS in any other ethnicity. We compared the association for these eight novel SNPs with type 2 diabetes in white Europeans from DIAGRAM. An overview of the study design is presented in Fig. 1a.

## Results

### Systematic review and meta-analysis

#### Search yields

A total of 3,898 articles were originally screened, of which 170 were carried to full-text review. Ninety-four studies met the inclusion and exclusion criteria, and 38 studies comprising 31 independent cohorts contained data on SNPs with evidence of genome-wide significance in any ethnicity. These 38 studies were included in the systematic review. Figure 1b depicts the selection process and lists reasons for exclusion at each stage. There was excellent agreement on study inclusion between reviewers (unweighted *κ* = 0.898).

#### Study characteristics

All included studies were in English and published in 2004 or later. Most large datasets (>2,500 people) were published after 2008. Eight of the 31 cohorts were from North India, four from South India, five from Pakistan, one from the Eastern region (Orissa), four from Indians residing in Singapore, two from Sri Lanka, one from Indians residing in Trinidad, one from Indians residing in Mauritius and one from all over the South Asian subcontinent; four were unspecified.

#### Participant characteristics

Studies eligible for analysis included 29,618 cases and 40,329 controls. Controls were described as healthy individuals who were normoglycaemic and/or non-diabetic. Men accounted for 54% of cases and 53% of controls. Age ranged from 34 to 62 years in cases [18, 19], and from 28 to 62 years in controls [18, 20]. BMI was reported for cases and controls in 79% of studies and ranged from 25.0 to 31.9 kg/m^2^ in cases [21, 22] and from 19.4 to 28.1 kg/m^2^ [23, 24] in controls. A majority of studies reported fasting glucose: 58% in cases (ranging from 6.41 to 10.60 mmol/l) [25, 26] and 63% in controls (ranging from 4.60 to 6.89 mmol/l) [14, 27]. Very few datasets reported additional clinical information; 29% reported waist circumference in cases and controls. Only three studies reported hip circumference. ESM Table 3 provides a summary of all included studies.

#### Quality assessment

Agreement with HWE in controls, genotyping call rate and source of controls and cases for included studies are summarised in ESM Table 4. One study did not report compliance with HWE [28]. SNPs in one study deviated from HWE [29]; rs2237892 and rs2237897 deviated from HWE at *p* = 0.002 and *p* = 0.001, respectively. The SNPs were excluded from analysis because the HWE threshold in the primary report was fairly conservative. Six studies did not appraise genotyping quality [28, 30–34]. Furthermore, three studies had a genotyping call rate below 95% for all reported SNPs [29, 35, 36].

#### Risk estimates for SNPs associated with type 2 diabetes in South Asians

**Fig. 2:**
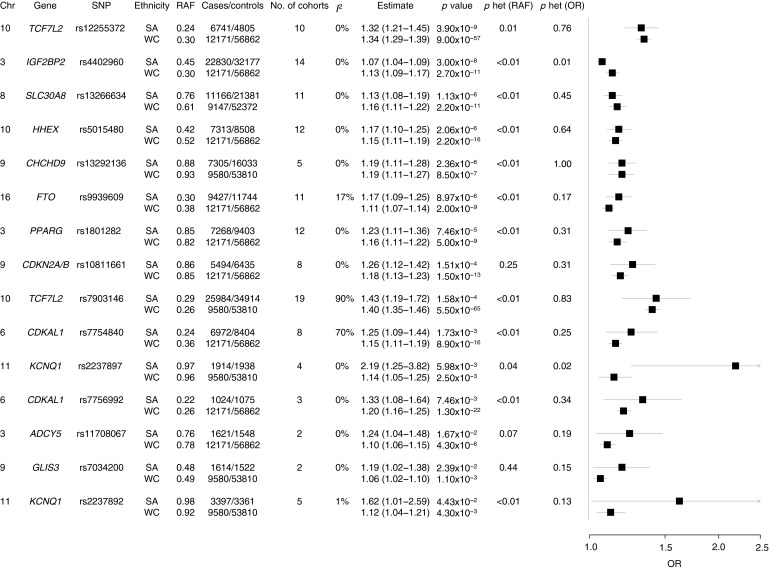
Forest plot of SNPs associated with type 2 diabetes in South Asians and comparison with white Europeans from DIAGRAM. Chr, chromosome; het, heterogeneity; no., number; SA, South Asian; WC, white European

Thirty SNPs with previous evidence of genome-wide significance in other ethnicities were available for meta-analysis. Pooled estimates were significant for 15 of the 30 SNPs (Fig. 2). Summary ORs, on the whole, suggested between a 1.15- and a 1.35-fold increase in susceptibility for type 2 diabetes per risk allele. Surprisingly, the risk alleles for both *KCNQ1* polymorphisms substantially increased the odds of type 2 diabetes (rs2237892 OR 1.62, 95% CI 1.01–2.59; rs2237897 OR 2.19, 95% CI 1.25–3.82). High degrees of between-study heterogeneity were evident for *CDKAL1* rs7754840 (*I* ^2^ = 70%, *Q* = 19.99, *p* < 0.01) and *TCF7L2* rs7903146 (*I* ^2^ = 90%, *Q* = 99.52, *p* < 0.01). In the *TCF7L2* meta-analysis, there was some evidence that larger studies had more conservative estimates, although Chauhan et al [35] and Uma Jyothi et al [25] were exceptions. The smallest study (*n* = 40 case/control pairs) depicted an association that was directionally inconsistent with the others [22]. Heterogeneity estimates did not change much without this outlier (*I* ^2^ = 90%, *Q* = 90.31, *p* < 0.01). Additional sources of heterogeneity could include consanguinity [34, 37]. One very obvious outlier existed in the meta-analysis for *CDKAL1* (OR 2.22, 95% CI 1.64–2.98) [37]. Heterogeneity estimates without this outlier diminished substantially (*I* ^2^ = 0%, *Q* = 2.80, *p* = 0.73). None of the outliers was excluded from our final analysis.

A majority of genes associated with type 2 diabetes from this meta-analysis (*ADCY5*, *CDKAL1*, *CDKN2A/B*, *HHEX*, *IGF2BP2*, *SLC30A8*, *TCF7L2* and *KCNQ1*) are involved in pancreatic beta cell function, while two are implicated in insulin sensitivity (*PPARG*) and adiposity (*FTO*). This is not surprising as insulin secretion is a more heritable trait than insulin action [38]. In addition to the above, eight novel SNPs were identified from South-Asian-only GWAS; these are discussed later in this paper.

### Comparison of effect sizes, RAF, and risk alleles between South Asians and white Europeans

**Fig. 3:**
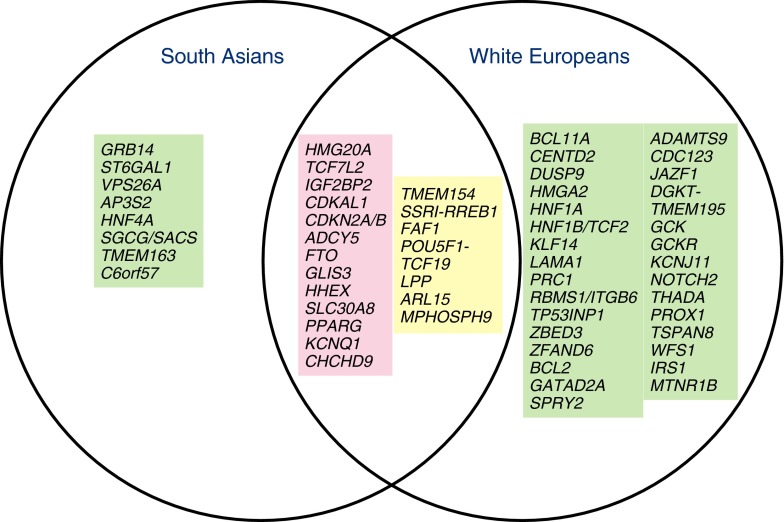
Venn diagram of SNPs common to South Asians and white Europeans and SNPs unique to both groups. *CENTD2* is also known as *ARAP1*. The green box includes genes with GWAS evidence for association with type 2 diabetes; the pink box includes genes with GWAS evidence in white Europeans and association with type 2 diabetes in this meta-analysis; and yellow with genes identified from a trans-ethnic meta-analysis of South Asians and white Europeans [49]

Meta-analysis estimates in South Asians were compared with white Europeans from DIAGRAM; no significant differences were identified for most SNPs (*p* < 0.05) (Fig. 2), although two SNPs showed heterogeneity (*KCNQ1* rs2237897 and *IGF2BP2* rs4402960). South Asians had a slightly smaller OR for *IGF2BP2* rs4402960 (South Asians: 1.07, 95% CI 1.04 − 1.09; white Europeans: 1.13, 95% CI 1.09 − 1.17). Figure 3 presents a Venn diagram of overlapping type 2 diabetes predisposing genes in South Asians and white Europeans, as well as genes unique to both groups.

Variation in RAFs between South Asians and white Europeans was observed, but no consistent trend was evident; South Asians did not consistently have an RAF greater than white Europeans (Fig. 2). While the risk allele for *HHEX* was the same in both ethnicities, the RAF differed; the risk allele is the minor allele in South Asians, but the major/common allele in white Europeans. Risk alleles for six SNPs (*SLC30A8* rs13266634, *ADCY5* rs11708067, *PPARG* rs1801282, *CHCHD9* (also known as *CHCHD2P9*) rs13292136, *KCNQ1* rs2237897 and *CDKN2A*/*B* rs10811661) were major/common alleles in both ethnicities.

### Testing novel SNPs discovered from South Asian GWAS in white Europeans

The systematic review included four GWAS in South Asians, which identified nine SNPs associated with type 2 diabetes at genome-wide significance. With the exception of *HMG20A* rs7178572 (South Asian OR: 1.09, 95% CI 1.06–1.12; white European OR: 1.08, 95% CI 1.05–1.10) [14, 39], the remaining eight SNPs have not been discovered in any other ethnic group. Five of the eight (*GRB14* rs3923113, *VPS26A* rs1082295, *HNF4α* [also known as *HNF4A*] rs4812829, *ST6GAL1* rs16861329, and *AP3S2* rs2028299) were independently replicated in South Asians [15].

We tested the eight novel SNPs for association with type 2 diabetes in DIAGRAM. *SGCG* rs9552911 was monomorphic in white Europeans. The remaining SNPs were directionally consistent with South Asian estimates and three (*AP3S2* rs2028299, *GRB14* rs3923113 and *HNF4α* rs4812829) were nominally (*p* < 0.05) associated with type 2 diabetes (Table 1) in DIAGRAM. The other four were not associated with type 2 diabetes in white Europeans. No consistent trend in RAFs was observed. The risk allele for *TMEM163* rs6723108 is the same in both ethnicities, but the RAF differs. Specifically, the risk allele is the minor allele in white Europeans, but the major/common allele in South Asians.

**Table 1:** Comparison of SNPs discovered from South Asian GWAS with white Europeans estimates from the DIAGRAM consortium

Chr.	Gene	SNP	Risk allele	South Asian					White European			*p*_heterogeneity_
				Reference	Sample size (cases/controls)	RAF	OR (95% CI)	Meta-analysed OR (95% CI)^a^	RAF^b^	DIAGRAM OR (95% CI)	Sample size (cases/controls)	
13	*SGCG*	rs9552911	G	Saxena et al, 2013 [16]	2620/4284	0.08	1.49 (1.30–1.72)	1.49 (1.30–1.72) (*p* = 4.25 × 10^–8^)	–	–	–	–
15	*AP3S2*	rs2028299	C	Kooner et al, 2011 [14]	18731/39856	0.31	1.10 (1.07–1.13)	1.10 (1.07–1.13) (*p* = 2.49 × 10^–11^)	0.26	1.04 (1.00–1.09) (*p* = 0.03)	9580/53810	0.03
				Tabassum et al, 2013 [15]	1256/1209	0.30	1.10 (0.96–1.25)					
3	*ST6GAL1*	rs16861329	C	Kooner et, 2011 [14]	18731/39856	0.75	1.09 (1.06–1.12)	1.09 (1.06–1.12) (*p* = 1.94 × 10^–9^)	0.87	1.03 (0.97–1.09) (*p* = 0.39)	6201/48359	0.09
				Tabassum et al, 2013 [15]	1256/1209	0.75	1.14 (0.99–1.31)					
6	*C6orf57*	rs1048886	G	Sim et al, 2011 [17]	977/1169	0.18	1.54 (1.32–1.80)	1.54 (1.32–1.80) (*p* = 8.32 × 10^–8^)	0.17	1.01 (0.97–1.06) (*p* = 0.56)	12171/56862	<0.01
2	*GRB14*	rs3923113	A	Kooner et al, 2011 [14]	18731/39856	0.74	1.09 (1.06–1.13)	1.09 (1.06–1.13) (*p* = 2.06 × 10^–7^)	0.61	1.04 (1.00–1.08) (*p* = 0.03)	9580/53810	0.07
				Tabassum et al, 2013 [15]	1256/1209	0.76	1.15 (0.99–1.33)					
15	*HMG20A*	rs7178572	G	Kooner et al, 2011 [14]	18731/39856	0.52	1.09 (1.06–1.12)	1.09 (1.06–1.12) (*p* = 1.94 × 10^–9^)	0.68	1.08 (1.05–1.10)^c^ (*p* = 2.17 × 10^–8^)	22669/58119	0.62
				Tabassum et al, 2013 [15]	1256/1209	0.51	1.15 (1.02–1.30)					
20	*HNF4α*	rs4812829	A	Kooner et al, 2011 [14]	18731/39856	0.29	1.09 (1.06–1.12)	1.13 (1.02–1.26) (*p* = 0.02)	0.16	1.07 (1.02–1.12) (*p* = 0.01)	9580/53810	0.35
				Tabassum et al, 2013 [15]	1256/1209	0.28	1.15 (1.02–1.30)					
10	*VPS26A*	rs1802295	T	Kooner et al, 2011 [14]	18731/39856	0.26	1.08 (1.05–1.12)	1.08 (1.06–1.10) (*p* = 3.02 × 10^–15^)	0.27	1.02 (0.98–1.06) (*p* = 0.28)	12171/56862	0.01
				Tabassum et al, 2013 [15]	1256/1209	0.27	1.09 (0.95–1.25)					
2	*TMEM163* ^d^	rs6723108	T	Tabassum et al, 2013 [15]	1256/1209	0.87	1.31 (1.20–1.44)	1.31 (1.20–1.44) (*p* = 1.26 × 10^–8^)	0.62	1.01 (0.97–1.04) (*p* = 0.71)	9580/53810	<0.01

^a^ORs are from meta-analysis of studies, where applicable

^b^RAF from 1,000 genomes (EUR)

^c^Estimate obtained from stage 2 (sample size is an approximate); found to be significantly associated with type 2 diabetes in white Europeans

^d^OR adjusted for age and sex

Chr., chromosome

### Population burden

**Fig. 4:**
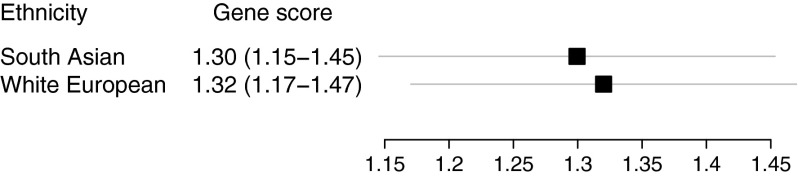
Gene score of SNPs in South Asians and white Europeans. The gene score was constructed using effect estimates and RAFs from SNPs common to both groups

All sixteen SNPs, which were significantly associated with type 2 diabetes in South Asians, were included in the genotype score (*ADCY5*, *CDKAL1* rs7754840 and rs7756992, *CDKN2A/2B*, *FTO*, *GLIS3*, *HHEX*, *PPARG*, *SLC30A8*, *TCF7L2* rs7903146 and rs12255372, *CHCHD9*, *KCNQ1* rs2237892 and rs2237897, *IGF2BP2* and *HMG20A* SNPs from Table 1 and Fig. 2). RAFs for both ethnicities were weighed by white European summary ORs from DIAGRAM because our primary analysis showed that individual risk estimates did not vary, and due to a larger sample, we expect the white European estimates to be more precise. Using this approach, no difference in the population burden was observed (*p* = 0.85) (Fig. 4).

## Discussion

Twenty-four SNPs were associated with type 2 diabetes in South Asians, eight of which were novel, discovered from GWAS. There were some variations in RAFs but the effect sizes for common SNPs did not differ between the ethnic groups. Interestingly, only three of the novel SNPs discovered from the South Asian GWAS were nominally associated with type 2 diabetes in white Europeans. Overall, the population burden from type 2 diabetes SNPs estimated using a genotype score appears to be comparable in both ethnicities.

### SNPs associated with type 2 diabetes from meta-analysis of South Asian studies

Meta-analysis of risk alleles for most SNPs increased the odds of type 2 diabetes by 15–35% among South Asians. Notable exceptions were the *KCNQ1* SNPs, which showed prominent ORs for type 2 diabetes. *KCNQ1* SNPs have been shown as a more significant contributor to type 2 diabetes than other loci in other Asian populations [40] and because both SNPs (in weak LD) with larger ORs are located on the same gene, it appears that *KCNQ1* may truly have a stronger signal in South Asians than in white Europeans. However, the exceptionally large effect size for *KCNQ1* rs2237897 is likely to be inaccurate, given the wide CIs and inconsistency with GWAS estimates from Europeans and East Asians (OR 1.33, 95% CI 1.24–1.41) [41]. Moreover, the *KCNQ1* rs2237897 association was reported in only two studies among South Asians and thus could be a product of the winner’s curse [42].

Fifteen GWAS signals for type 2 diabetes in white Europeans, East Asians, and Singaporean Malay [43–46] were not associated with type 2 diabetes in our South Asian meta-analysis. This may reflect low power to detect similar effect sizes for *ADAMTS9* rs4607103, *CDC123* rs12779790, *JAZF1* rs864746, *KCNQ1* rs231362, *DGKT-TMEM195* rs2191349 (also known as *AGMO*), *GCK* rs1799884, *GCKR* rs780094, *MTNR1B* rs10830963, *PROX1* rs340874, and *TSPAN8* rs7961581 (Table 2) and either a true lack of association for *KCNJ11* rs5219, *KCNQ1* rs2237895, *NOTCH2* rs10923931, *THADA* rs7578597 and *WFS1* rs10010131 or significant between-study heterogeneity since we were adequately powered for the last five SNPs (Table 2). *KCNJ11* rs5219 in particular was close to significance (OR 1.19. 95% CI 0.98–1.45) and demonstrated a high degree of heterogeneity (*I* ^2^ = 81%, *p* < 0.01).

**Table 2:** SNPs associated with type 2 diabetes in published GWAS from white Europeans but not replicated in South Asian meta-analysis

Chr.	Gene	SNP	GWAS estimate		South Asian meta-analysis		Direction^c^	Cases in current meta-analysis	Power in this meta-analysis	Case–control pair needed for 80% power
			Reference	OR (95% CI)^a^	OR (95% CI)^b^	RAF				
3	*ADAMTS9*	rs4607103	Zeggini et al, 2008 [44]	1.09 (1.06–1.12)	1.01 (0.90–1.14)	0.50	+	2307	0.54	4231
10	*CDC123*	rs12779790	Zeggini et al, 2008 [44]	1.11 (1.07–1.14)	1.11 (0.97–1.27)	0.14	+	4049	0.65	6135
7	*JAZF1*	rs864745	Zeggini et al, 2008 [44]	1.10 (1.07–1.13)	1.08 (0.96–1.22)	0.71	+	4055	0.78	4285
11	*KCNQ1*	rs2237895	Tsai et al, 2010 [43]	1.29 (1.19–1.40)	1.12 (0.96–1.31)	0.42	+	1424	0.99	496
7	*DGKT –TMEM195*	rs2191349	Dupuis et al, 2010 [47]	1.06 (1.04–1.08)	1.04 (0.85–1.27)	0.61	+	1632	0.21	9771
7	*GCK*	rs1799884	Dupuis et al, 2010 [47]	1.07 (1.05–1.10)	0.92 (0.73–1.15)	0.14	+	1670	0.16	13,971
7	*GCKR*	rs780094	Dupuis et al, 2010 [47]	1.06 (1.04–1.08)	1.07 (0.96–1.19)	0.76	+	4055	0.35	12,833
11	*KCNJ11*	rs5219	Scott et al, 2007 [48]	1.14 (1.10–1.19)	1.19 (0.98–1.45)	0.37	+	5643	1.00	1939
7	*PROX1*	rs340874	Dupuis et al, 2010 [47]	1.07 (1.05–1.09)	0.96 (0.83–1.11)	0.58	+	1626	0.27	7073
11	*MTNR1B*	rs10830963	Dupuis et al, 2010 [47]	1.09 (1.06–1.12)	1.01 (0.91–1.11)	0.40	+	3475	0.70	4377
2	*THADA*	rs7578597	Zeggini et al, 2008 [44]	1.15 (1.10–1.20)	1.05 (0.92–1.20)	0.86	+	4090	0.86	3313
11	*KCNQ1*	rs231362	Voight et al, 2010 [45]	1.08 (1.06–1.10)	1.15 (0.97–1.36)	0.75	+	3052	0.45	6847
1	*NOTCH2*	rs10923931	Zeggini et al, 2008 [44]	1.13 (1.08–1.17)	1.06 (0.94–1.19)	0.21	+	4035	0.89	3581
12	*TSPAN8*	rs7961581	Zeggini et al, 2008 [44]	1.09 (1.06–1.12)	1.05 (0.95–1.16)	0.33	+	3977	0.73	4865
4	*WFS1*	rs10010131^d^	Voight et al, 2010 [45]	1.13 (1.08–1.18)	1.05 (0.96–1.15)	0.72	+	5022	0.97	2472

^a^ORs are from the referenced GWAS

^b^ORs are from the South Asian meta-analysis

^c^+ is the same direction of effect, − is the opposite direction of effect

^d^GWAS significant SNP rs1801214 is in LD (*r* ^2^ = 1) with reported SNP

Chr., chromosome

### Comparison of effect sizes, RAF and risk alleles between South Asians and white Europeans

In general when comparing effect sizes of SNPs associated with type 2 diabetes from our meta-analysis with DIAGRAM estimates in white Europeans, the risk from SNPs predisposing to type 2 diabetes did not differ substantially between the groups. However, the point estimates were more precise and CIs tighter among the white Europeans because of the larger sample size. Our observation of no significant difference in risk estimates is not surprising because SNPs evaluated in South Asians are selected for homogeneity as they were first discovered in white Europeans and then replicated in South Asians. If there is a difference in genetic risk between the ethnic groups, it probably does not result from polymorphisms common to both groups. The results of our paper are supported by those recently published by DIAGRAM, the Asian Genetic Epidemiology Network Type 2 Diabetes (AGEN-T2D) Consortium, and the South Asian Type 2 Diabetes (SAT2D) Consortium [49]. Their study also found effect estimates for common SNPs predisposing to type 2 diabetes to be homogenous among South Asians and white Europeans. In addition to the recently published study, we show that the gene score, which measures population burden in both groups, does not differ. Our conclusion that the genetic risk for type 2 diabetes probably does not differ between the two ethnicities is greatly strengthened by this recent publication.

Figure 3 depicts overlap between genes associated with type 2 diabetes in South Asians and white Europeans. Considerably more signals have been identified in white Europeans due to the greater number of GWAS. Because a majority of signals were replicated in our meta-analysis, it is unlikely that they are unique to white Europeans. Rather, larger GWAS with greater than 36,000 case/control pairs are required to detect ORs as low as 1.05 with an RAF of 10%. The four GWAS in South Asians represent about 25,704 cases and 43,688 controls, therefore more GWAS in South Asians alone will detect small effects and discover SNPs unique to this group, and ultimately facilitate further elucidation of the genetic basis of type 2 diabetes in this group.

We did not find a trend in RAFs; specifically, RAFs were not consistently higher in South Asians. Risk alleles from six SNPs in this analysis were the major/common alleles in both ethnicities. Negative selection usually prevents risk alleles from becoming common unless they are advantageous [50]. In the case of *PPARG* rs1801282, the risk allele is responsible for increased fat storage and may have been advantageous in ancient environments with unpredictable food supply and high level of activity, but now predisposes to type 2 diabetes [50]. However, to truly establish whether active selection of an allele exists, systematic study of population differentiation is warranted [51].

### Testing novel SNPs discovered from South Asians GWAS in white Europeans

Finally, we tested novel SNPs derived from South Asians in white Europeans; only three were associated with type 2 diabetes. Interestingly, one of the novel SNPs, rs1048886, is a functional missense mutation resulting in a change from glutamine to arginine in the UPF0369 protein involved in immune response [52]. Additionally, the G risk allele for *SGCG* rs9552911 is not present in white Europeans. The putative existence of private polymorphisms in South Asians is supported by presence of assortative mating resulting from prolonged geographical and cultural isolation of this region from white Europeans [53]. Non-association of the remaining seven dimorphic SNPs with type 2 diabetes in white Europeans is quite puzzling as the risk alleles are present in this group. DIAGRAM’s large sample ensures reasonable power; for the lowest RAF of all eight SNPs, the DIAGRAM sample had greater than 80% power to detect an OR of at least 1.07, and therefore non-association may be due to substantially different LD structures or lower effect sizes in white Europeans because of ethnic specific gene–environment or gene–gene interactions. We compared the LD structure for the seven SNPs using HapMap data, and while not substantial, *r* ^2^ values with neighbouring SNPs have some differences (for example, *r* ^2^ of rs1048886 with neighbouring rs9455158 is 0.75 in white Europeans and 0.87 in South Asians; *r* ^2^ of rs3923113 with neighbouring rs13432797 is 0.93 in white Europeans and 0.64 in South Asians). Fine mapping analyses will determine whether the same functional variants are responsible for increased risk in both groups and if the effect is comparable, or if the seven SNPs tag unique functional variants in South Asians.

### Population burden

No difference in genotype score between South Asians and white Europeans was observed, which is consistent with our finding that no trend exists for variation in RAFs. It should be noted that our conclusion is based on an assumption of homogeneity in effect sizes between the groups, supported by our primary analysis. Our conclusion is not consistent with some literature, which shows a greater population burden in South Asians based on unweighted genotype scores [54, 55]. However, while the cumulative genotype score for South Asians was statistically higher than white Europeans in the referenced study, the magnitude of the difference is small (0.99 points on a scale that varied from 0 to 32) [54]. Moreover, unweighted scores consider the contribution from SNPs with larger effects to be the same as that from SNPs with small effects, and appear to discriminate less effectively between disease states [56–59]. It should be noted, though, that the genotype score calculation in the referenced paper is much more direct than ours as it is based on primary data.

### Strengths and limitations

This is the first meta-analysis that compares genetic risk of type 2 diabetes in South Asians and white Europeans. Potential limitations of our study include use of unadjusted allelic OR, which precluded us from accounting for SNP–type 2 diabetes associations altered by adiposity. Use of unadjusted allelic OR depended largely on the unavailability of appropriately adjusted data. However, we informally compared adjusted estimates reported in the study with our unadjusted allelic OR and found the adjustment to make little difference; for example, the OR for *GCKR* rs780094 in the primary report [55] was 0.87 (95% CI 0.72–1.05) after adjustment for age and sex. In our study, the OR is 0.86 (95% CI 0.65–1.13).

Second, publication and time-lag biases may exist whereby significant results are published more often than negative studies. To minimise these biases, we conducted a thorough search of literature and consulted experts to identify as many eligible studies as possible. We were unable to produce funnel plots to formally assess publication bias because fewer than ten studies were used to meta-analyse a majority of SNPs.

Third, some SNPs in this meta-analysis were only investigated in two cohorts and their summary estimates should be interpreted with caution.

Finally, sample sizes varied with each SNP in South Asians but were consistent for all SNPs in white Europeans, which may create artificial differences between the groups as a result of differences in precision. Our conclusions regarding the genetic heterogeneity between South Asians and white Europeans are applicable only to genetic risk from bi-allelic common SNPs as our investigation was limited to this type of variant.

### Future directions

The question of why South Asians have a greater risk for type 2 diabetes remains unanswered from a genetic perspective as risk from common SNPs predisposing to type 2 diabetes does not differ. Our results are particularly important as they emphasise the need for future research to explore heritable epigenetic changes, epistasis and gene–environment interactions to answer this question [60, 61], rather than focusing on exploring differences in risk estimates and RAFs. Additionally, differences in genetic risk may result from low-frequency causal variants with large effects (rare variants) [62, 63]. The existence of such rare variants can be investigated through candidate gene, whole-genome or whole-exome sequencing. In fact, discovery of rare variants for Crohn’s disease greatly supports this paradigm [64]. Large-scale exon resequencing around an *MTNR1B* SNP modestly associated with type 2 diabetes (OR between 1.10 and 1.15) identified four rare variants that led to loss of melatonin binding and signalling capacity (OR 5.67, 95% CI 2.17–14.82) [65]. Using a family-based approach with multiple affected members or studying founder populations can facilitate the identification of rare variants in sequencing studies. Finally, systematic reviews and primary studies comparing dietary intake and physical activity patterns between South Asians and white Europeans have noted considerable variation [66, 67], which may also contribute to the differences observed between the two ethnic groups.

## Conclusions

Similar effect sizes for SNPs predisposing to type 2 diabetes are apparent among South Asians and white Europeans, but there is variation in RAFs. Additionally, some novel SNPs are present in South Asians. Given the current literature, there is no strong evidence to indicate that currently known genetic variants explain the higher risk of type 2 diabetes in South Asians compared with white Europeans.

## Electronic supplementary material

Below is the link to the electronic supplementary material.

ESM Table 1 (PDF 43 kb)

ESM Table 2 (PDF 51 kb)

ESM Table 3 (PDF 168 kb)

ESM Table 4 (PDF 113 kb)

## Abbreviations

DIAGRAM: DIAbetes Genetics Replication And Meta-analysis
GWAS: Genome-wide association studies
HWE: Hardy–Weinberg equilibrium
LD: Linkage disequilibrium
RAF: Risk allele frequency
SNP: Single nucleotide polymorphism
