# Supplementary material for: Does genetic heterogeneity account for the divergent risk of type 2 diabetes in South Asian and white European populations?
Source: Diabetologia. 2014 Aug 22;57(11):2270–81. doi: 10.1007/s00125-014-3354-1 (PMC4180911; doi:10.1007/s00125-014-3354-1)
Supplement: Supplementary file 1 — (PDF 43 kb) [file 125_2014_3354_MOESM1_ESM.pdf]

Electronic Supplementary Material Table 1 – Full search strategy

1. diabetes.mp. or exp Diabetes Mellitus, Type 2/
2. exp Phenotype/ or exp Genetic Variation/ or exp Genetic Predisposition to Disease/ or exp Genotype/ or genetic variants.mp. or exp Polymorphism, Single Nucleotide/
3. exp Asian Continental Ancestry Group/ or south asian.mp.
4. ASIA$.mp.
5. INDIA$.mp. or India/
6. BANGLADESH$.mp. or Bangladesh/
7. PAKISTAN$.mp. or Pakistan/
8. SRI LANKA$.mp. or Sri Lanka/
9. CEYLON$.mp. or Sri Lanka/
10. exp India/ or india*.mp.
11. bengali*.mp.
12. indo*.mp.
13. gujarat*.mp.
14. sikh*.mp.
15. sind*.mp.
16. genome wide association study.mp. or Genetic Markers/ or exp Genome-Wide Association Study/ or Disease Susceptibility/ or Pedigree/ or Genetic Linkage/ or Genome, Human/
17. candidate gene*.mp.
18. exp Mutation/ or mutation.mp.
19. (south adj2 asia*).mp. [mp=title, abstract, original title, name of substance word, subject heading word, protocol supplementary concept, rare disease supplementary concept, unique identifier]
20. niddm.mp.
21. type 2 diabetes.mp.
22. genetic predisposition.mp. or exp Genetic Predisposition to Disease/
23. polymorphism.mp.
24. genotype.mp.
25. allelic frequenc*.mp.
26. genetic linkage.mp.
27. genetic marker*.mp.
28. phenotype.mp.
29. genetic varia*.mp.
30. single nucleotide polymorphism.mp.
31. punjab*.mp.
32. tamil.mp.
33. goa.mp.
34. kerala.mp.
35. sikkim.mp.
36. haryana.mp.
37. 2 or 16 or 17 or 18 or 22 or 23 or 24 or 25 or 26 or 27 or 28 or 29 or 30

38. rajasthan*.mp.
39. bihar*.mp.
40. kashmir*.mp.
41. maharashtra*.mp.
42. pradesh*.mp.
43. bharat*.mp.
44. 3 or 4 or 5 or 6 or 7 or 8 or 9 or 10 or 11 or 12 or 13 or 14 or 15 or 19 or 31 or 32 or 33 or 34 or 35 or 36 or 38 or 39 or 40 or 41 or 42 or 43
45. type 2 diabet*.mp.
46. 1 or 20 or 21 or 45
47. 37 and 44 and 46
48. limit 47 to animals
49. 47 not 48
