# Supplementary material for: Does genetic heterogeneity account for the divergent risk of type 2 diabetes in South Asian and white European populations?
Source: Diabetologia. 2014 Aug 22;57(11):2270–81. doi: 10.1007/s00125-014-3354-1 (PMC4180911; doi:10.1007/s00125-014-3354-1)
Supplement: Supplementary file 2 — (PDF 51 kb) [file 125_2014_3354_MOESM2_ESM.pdf]

Electronic Supplementary Material Table 2 - Reported SNPs and their proxies in linkage disequilibrium ($r^2 > 0.8$) determined using SNAP

Gene	Reported SNP	Lead SNP	r^2
CDKAL1	rs6931514	rs7756992	0.917
	rs10946398	rs7754840	1
	rs9295474	rs7754840	0.885
FTO	rs1421085	rs9939609	0.901
	rs7193144	rs9939609	0.967
	rs8050136	rs9939609	1
	rs9930506	rs9939609	0.839
	rs3751812	rs9939609	1
HHEX	rs1111875	rs5015480	0.966
HNF4A	rs1884613	rs4812829	0.848
	rs2144908	rs4812829	1
	rs1884614	rs4812829	1
KCNJ11	rs5215	rs5219	0.935
KCQN1	rs2283228	rs2337892	0.867
SLC30A8	rs11558471	rs13266634	0.960
TCF7L2	rs4506565	rs7903146	0.892
	rs7901695	rs7903146	0.892
IGF2BP2	rs1470579	rs4402960	1
CHCHD9	rs4295736	rs13292136	1
WFS1	rs1801214	rs10010131	1
