# Supplementary material for: Does genetic heterogeneity account for the divergent risk of type 2 diabetes in South Asian and white European populations?
Source: Diabetologia. 2014 Aug 22;57(11):2270–81. doi: 10.1007/s00125-014-3354-1 (PMC4180911; doi:10.1007/s00125-014-3354-1)
Supplement: Supplementary file 3 — (PDF 168 kb) [file 125_2014_3354_MOESM3_ESM.pdf]

Electronic Supplementary Material Table 3 – Characteristics of included studies

Reference (Author Journal Name Year)	Region of origin	N cases	N controls	Mean age cases	Mean age controls	Mean fasting glucose cases (mmol/l)	Mean fasting glucose controls (mmol/l)	Mean BMI cases	Mean BMI controls	Mean waist circumference cases	Mean waist circumference controls	Mean hip circumference cases	Mean hip circumference controls	WHR cases	WHR controls	% males cases	% males controls
Tai J Lipid Res 2004 [1]	Singaporean Indians	108	305	-	-	-	-	-	-	-	-	-	-	-	-	-	-
Radha Diabetes Care 2006 [2]	Not specified (US cohort)	81	616	56 (10)	42 (13)	8.93 (3.6)	5.33 (1.3)	25.6 (3.7)	24.9 (3.7)	-	-	-	-	-	-	-	-
	South India	799	820	52 (11)	41 (13)	8.99 (3.8)	4.72 (0.4)	25.1 (4.2)	23.4 (4.7)	-	-	-	-	-	-	-	-
Humphries J Mol Med 2006 [3]	Not specified	841	302	-	-	-	-	-	-	-	-	-	-	-	-	-	-
Chandak Diabetologia 2007 [4]	North India	955	399	47.2 (9.3)	30.9 (5.1)	-	5.1 (4.7–5.5)*	Men: 25.4 (3.6) Women: 27.1 (3.9)	Men: 20.5 (3.1) Women: 19.1 (2.5)	-	-	-	-	Men: 0.98 (0.06), women: 0.89 (0.06)	Men: 0.90 (0.06), women: 0.76 (0.05)	53.7	46.6
Bodhini Clin Exp Met 2007 [5]	South India	1031	1038	49 (10)	41 (11)	9.2 (4.0)	4.6 (0.4)	25.1 (4.3)	23.6 (4.6)	90.4 (10.0)	83.7 (11.6)	-	-	-	-	-	-
Sanghera BMC Med Genet 2008 [6]	North India	532	386	Men: 53.2 (11.1), Women: 55.2 (11.0)	Men: 51.7 (15.6), Women: 50.8 (13.0)	Men: 10.1 (3.7) Women: 10.0 (3.5)	Men: 5.4 (0.6) Women: 5.4 (0.5)	Men: 26.6 (4.3); Women: 29.0 (5.5)	Men: 26.6 (4.4), Women: 27.5 (4.9)	-	-	-	-	Men: 0.99 (0.06), Women: 0.93 (0.07)	Men: 0.97 (0.07), Women: 0.91 (0.07)	56.2	47.7
Sanghera Ann Hum Genet 2008 [7]	North India	556	537	Men: 56.22 (10.9), Women: 55.05 (11.0)	Men: 52.58 (15.7), Women: 51.63 (13.0)	Men: 9.89 (3.62), Women: 10.0 (3.45)	Men: 5.36 (0.56), Women: 5.40 (0.57)	Men: 26.42 (4.3), Women: 29.10 (5.5)	Men: 26.65 (4.4), Women: 27.40 (4.9)	-	-	-	-	Men: 0.99 (0.06), Women: 0.93 (0.07)	Men: 0.97 (0.07), Women: 0.90 (0.07)	55.6	48.4
Rees BMC Med Genet 2008 [8]	Pakistan (Punjab)	831	437	56.9 (12.1)	55.0 (11.8)	-	-	28.3 (4.7)	28.1 (4.9)	102.4 (10.7)	99.8 (13.1)	-	-	-	-	-	-
Sanghera J Human Genet 2009 [9]	North India (Punjab)	680	637	men: 55.8 (11.2) women: 56.9 (10.8)	men: 51.1 (15.1), women: 50.2 (13.2)	men: 10.3 (3.7), women: 10.6 (3.8)	men: 5.4 (0.7), women: 5.4 (0.7)	men: 26.5 (4.7), women: 28.0 (5.0)	men: 26.3 (4.5), women: 27.0 (5.1)	men: 94.8 (10.5), women: 92.8 (11.4)	men: 92.3 (11.5), women: 87.6 (10.8)	men: 96.0 (8.4), women: 99.5 (10.3)	men: 96.2 (8.8), women: 97.0 (10.2)	men: 0.99 (0.31), women: 0.93 (0.15)	men: 0.96 (0.09), women: 0.90 (0.16)	-	-

Yajnik Diabetologia 2009 [10]	North India	1453	1361	46.6 (9.3)	34.5 (6.1)	8.50 (6.89-11.28)*	5.06 (4.61-5.56)*	Men: 25.4 (4.0), Women: 27.2 (4.3)	Men: 21.9 (3.6), Women: 20.9 (4.1)	Men: 95.4 (10.7), Women: 92.4 (10.3)	Men: 83.2 (10.5), Women: 69.9 (10.4)	Men: 97.3 (7.4), Women: 103.2 (10.2)	Men: 91.6 (7.5), Women: 90.9 (9.3)	Men: 0.98 (0.06), Women: 0.89 (0.06)	Men: 0.91 (0.06), Women: 0.77 (0.06)	56.3	53.6
Haseeb J Biosci 2009 [11]	South India	350	349	61.8 (11.25)	61.93 (10.40)	-	-	-	-	-	-	-	-	-	-	-	-
Chauhan Diabetes 2010 [12]	North India (Pune)	1467	1672	46 (40–52)*	33 (29–37)*	8.50 (6.90–11.30)*	5.11 (4.67–5.56)*	Men: 24.90 (22.80–27.70), Women: 26.90 (24.40–29.60)*	Men: 21.18 (19.15–23.62), Women: 19.53 (17.60–22.74)*	-	-	-	-	Men: 0.97 (0.94-1.02), Women: 0.89 (0.85-0.94)*	Men: 0.91 (0.86-0.95), Women: 0.76 (0.73-0.80)*	56.3	52.9
	North India (Delhi)	1019	1006	53 (45–62)*	50 (44–60)*	7.90 (6.40–10.30)*	4.90 (4.50–5.30)*	Men: 23.80 (22.00–26.00), Women: 26.70 (24.20–29.20)*	Men: 23.20 (20.20–25.70), Women: 24.90 (21.10–28.60)*	-	-	-	-	Men: 1.00 (0.97–1.03), Women: 1.00 (0.97–1.03)*	Men: 0.97 (0.92–1.00), Women: 0.86 (0.82–0.92)*	58.1	60.2
Gupta Ann Hum Genet 2010 [13]	North India	219	184	Men: 59.12 (10.34), Women: 55.65 (10.44)	Men: 55.52 (10.61), Women: 51.99 (9.37)	-	-	Men: 27.18 (5.75), Women: 30.08 (4.91)	Men: 27.48 (7.00), Women: 29.64 (5.27)	-	-	-	-	Men: 1.01 (0.09), Women: 0.92 (0.08)	Men: 1.00 (0.08), Women: 0.89 (0.07)	66.2	51.6
Chidambaram Metabolism 2010 [14]	South India	926	812	52 (11)	38 (12)	8.97 (4.13)	4.68 (0.44)	25 (4)	23 (4)	-	-	-	-	-	-	-	-
Mukhopadhyaya Genet Mol Res 2010 [15]	North India	40	40	Men: 46.0 (14.6), Women: 48.6 (9.0)	Men: 42.29 (13.41), Women: 45 (8.83)	Men: 10.25 (1.88), Women: 10.28 (1.47)	Men: 5.12 (0.94); Women: 5.14 (0.73)	Men: 31.9 (5.0), Women: 31.9 (4.0)	Men: 27.9 (4.9), Women: 28.4 (4.3)	-	-	-	-	Men: 0.99 (0.06), Women: 1.01 (0.08)	Men: 0.97 (0.06), Women: 0.99 (0.08)	52.5	52.5
Sanghera Metabolism 2010 [16]	North India	554	527	Men: 56.1 (11.1), Women: 55.2 (11.1)	Men: 51.8 (15.6), Women: 51.5 (13.3)	Men: 9.4 (3.3), Women: 9.6 (3.1)	Men: 5.4 (0.6), Women: 5.5 (0.6)	Men: 26.6 ± 4.4; Women: 29.0± 5.4	Men: 26.7 (4.2), Women: 27.5 (4.8)	-	-	-	-	Men: 0.99 (0.08), Women: 0.93 (0.07)	Men: 0.97 (0.08), Women: 0.91 (0.07)	55.8	49.0
Vimaleswaran Met 2010 [17]	South India	1000	1000	52 (11)	46 (12)	-	-	26.1 (4.2)	24.0 (4.7)	92.3 (9.4)	87.2 (11.4)	-	-	-	-	-	-

Tan J Clin End 2010 [18]	Singaporean Indians	246	364	-	-	-	-	-	-	-	-	-	-	-	-	-	-
Rees Diabet Med 2011 [19]	Pakistan	385	1281	53.5 (10.7)	51.1 (10.7)	10.6 (4.0)	5.3 (0.6)	26.7 (5.6)	25.2 (5.2)	93.2 (11.7)	88.1 (12.0)	-	-	-	-	40.0	46.3
	North India and Pakistan	1568	1177	55.8 (11.9)	56.4 (10.7)	-	5.4 (0.6)	27.5 (4.8)	25.7 (5.2)	99.8 (11.4)	93.8 (13.0)	-	-	-	-	51.6	50.0
Rees PloS One 2011 [20]	Pakistan	857	417	56.9 (12.0)	54.9 (11.7)	-	-	28.6 (4.6)	28.0 (4.9)	-	-	-	-	-	-	45.3	52.0
	Pakistan	821	1167	54.6 (11.7)	56.3 (10.8)	-	5.5 (0.6)	26.1 (4.7)	24.3 (5.0)	-	-	-	-	-	-	52.4	52.9
Chavali J Human Genet 2011 [21]	North India	1019	1006	53 (45-62)*	50 (44-60)*	7.9 (6.4-10.3)*	4.9 (4.5-5.3)*	Men: 23.80 (22.00-26.00), Women:26.70 (24.20-29.20)*	median: men=23.10 (20.10-25.70), women=25.00 (21.10-28.50)	-	-	-	-	Men: 1.00 (0.97-1.03), Women: 1.00 (0.97-1.03)*	Men: 0.97 (0.92-1.00), Women: 0.86 (0.82-0.92)*	58.1	60.2
Boodram West Indian Med J 2011 [22]	Indo-Trinidadian	168	61	45.11 (12.03)	51.68 (17.22)	-	-	26.05 (4.47)	25.08 (5.03)	-	-	-	-	-	-	27.4	40.5
Rees Diabetologia 2011 [23]	Pakistan	1678	1584	UKADS: 56.90 (12.30), DGP: 54.62 (11.67)	UKADS: 54.92 (11.75), DGP: 56.27 (10.81)	-	DGP: 4.75 (0.47)	UKADS: 28.56 (4.61), DGP: 26.07 (4.72)	UKADS: 28.01 (4.86), DGP: 24.30 (5.03)	UKADS: 102.31 (10.48), DGP: 96.78 (11.88)	UKADS: 99.75 (11.52), DGP: 91.91 (13.13)	-	-	-	-	UKADS: 54.7%, DGP: 47.6%	UKADS: 47.96%, DGP: 47.13%
Sim PLoS Genet 2011 [24]	Singaporean Indians	977	1169	60.71 (9.85)	55.73 (9.72)	-	-	27.06 (5.10)	25.33 (4.40)	-	-	-	-	-	-	54.4	48.4
Chauhan J Hum Genet 2011 [25]	North India (replication cohort)	1401	1848	55 (48–62)*	52 (45–62)*	8.05 (6.39–10.60)*	4.82 (4.41–5.20)*	Men: 25.40 (22.94–28.36), Women: 27.34 (24.46–31.00)*	Men: 24.69 (22.15–27.35), Women: 26.30 (23.11–29.23)*	Women: 86.68 (40.00-98.00), Men: 90.00 (36.00-98.00)*	Women: 88.00 (81.00-95.50), Men: 93.00 (86.00-100.00)*	-	-	Women: 0.95 (0.89-0.98), Men: 0.98 (0.96-1.03)*	Men: 0.97 (0.92-1.00), Women: 0.87 (0.82-0.91)*	60.7	55.0

	North India	1019	1006	53 (45-62)*	50 (44-60)*	7.90 (6.40–10.30)*	4.90 (4.50–5.30)*	Men: 23.80 (22.00–26.00), Women: 26.70 (24.20–29.20)*	Men: 23.20 (20.20–25.70), Women: 24.90 (21.10–28.60)*	Men: 86.36 (86.36–91.44), Women: 91.44 (86.36–96.52)*	Men: 88.50 (80.64–95.00), Women: 85.00 (75.60–93.00)*	-	-	Men: 1.0 (0.97-1.03), Women: 1.0 (0.97-1.03)*	Men: 0.97 (0.92–1.00), Women: 0.86 (0.82–0.92)*	58.1	60.2
Anuradha Clin Genet 2011 [26]	South India (Chennai)	792 (505 Early onset) (287 Late onset)	247	Early onset: 34 (4), Late onset: 52 (7)	58 (7)	Early onset: 9.5 mmol (3.9), Late onset: 8.5 (3.6)	4.7 (0.4)	Early onset: 25.7 (4.2), Late onset: 24.8 (4.3)	22.4 (0.7)	-	-	-	-	-	-	-	-
Kooner Nat Genet 2011 [27]		18731	39856	LOLIPOP 610: 59.4 (9.2); LOLIPOP 317: 54.1 (10.1); SINDI: 60.7 (9.9); PROMIS: 55.0 (9.4)	LOLIPOP 610: 53.9 (10.7); LOLIPOP 317: 46.8 (10.1); SINDI: 55.7 (9.7); PROMIS: 52.9 (10.5)	LOLIPOP 610: 8.6 (3.1); LOLIPOP 317: 5.2 (0.6); SINDI: 8.9 (2.9); PROMIS: 9.71 (4.44)	LOLIPOP 610: 5.2 (0.6); LOLIPOP 317: 5.1 (0.6); SINDI: 5.38 (1.06); PROMIS: 6.89 (2.91)	LOLIPOP 610: 28.1 (4.6); LOLIPOP 317: 27.6 (4.7); SINDI: 27.1 (5.1); PROMIS: 26.0 (4.0)	LOLIPOP 610: 26.8 (4.2); LOLIPOP 317: 26.6 (4.2); SINDI: 25.3 (4.4); PROMIS: 25.3 (3.9)	LOLIPOP 610: 100.8 (11.5); LOLIPOP 317: 100.0 (12.2); SINDI: - ; PROMIS: 92.0 (12.0)	LOLIPOP 610: 96.6 (10.9); LOLIPOP 317: 96.3 (11.4); SINDI: - ; PROMIS: 90.1 (11.7)	-	-	LOLIPOP 610: 0.99 (0.07); LOLIPOP 317: 0.99 (0.07); SINDI: - ; PROMIS: 0.95 (0.06)	LOLIPOP 610: 0.95 (0.07); LOLIPOP 317: 0.95 (0.07); SINDI: - ; PROMIS: 0.94 (0.07)	LOLIPOP 610: 82.9; LOLIPOP 317: 100.0; SINDI: 54.4; PROMIS: 76.5	LOLIPOP 610: 84.8; LOLIPOP 317: 100.0; SINDI: 48.4; PROMIS: 83
Been BMC Med Genet 2011 [28]	Not specified (US cohort)	139	557	-	-	8.10 (2.4)	5.47 (0.7)	-	-	-	-	-	-	-	-	-	-
	North India	1290	1019	-	-	9.03 (3.5)	5.27 (0.74)	-	-	-	-	-	-	-	-	-	-
Ramya Diabetes Technol Ther 2011 [29]	South India	851	1001	51 (11)	41 (13)	9.0 (3.9)	4.7 (0.4)	25.3 (4.3)	23.4 (4.7)	91.0 (10.1)	83.5 (12.1)	98.0 (10.0)	94.0 (10.0)	-	-	44.3	41.8
Janipali Diabetic Med 2012 [30]	North India	1808	1549	51.8 (45.6-58.6)	40.0 (36.1-44.4)	8.2 (6.7-11.0)	5.1 (4.8-5.5)	25.7 (23.4-28.6)	19.4 (17.7-21.7)	-	-	-	-	0.90 (0.88-0.99)	0.84 (0.77-0.91)	55.8	53.2

Been Nutr Metab 2012 [31]	North India	1201	1021	Men: 54.1 (10.2), Women: 53.7 (9.8)	Men: 51.3 (15.2), Women: 50.1 (13.3)	Men: 9.09 (3.4), Women: 9.06 (3.6)	Men: 5.29 (0.6), Women: 5.20 (0.6)	Men: 26.6 (4.4), Women: 28.4 (5.4)	Men: 25.7 (4.8), Women: 27.1 (6.7)	-	-	-	-	-	-	52.3	52.4
Raza Gene 2012 [32]	North India	87	88	48.47 (12.16)	33.79 (12.07)	-	-	25.80 (3.97)	23.35 (3.02)	-	-	-	-	-	-	67.8	53.4
Anand Diabetes Care 2013 [33]	Not specified	638	2125	47.72 (9.40)	44.14 (9.22)	7.35 (2.87)	4.80 (0.78)	27.0 (4.1)	26.3 (4.4)	100.9 (10.2)	100.1 (10.4)	-	-	-	-	40.5	50.8
Ali PLoS One 2013 [34]	North India	1583	1317	53.28 (10.13)	51.47 (12.40)	8.82 (3.36)	4.64 (0.66)	25.86 (4.76)	24.82 (4.35)	-	-	-	-	-	-	-	-
Sexena Diabetes 2013 [35]		19482	27821							-	-	-	-	-	-	-	-
Tabassum Diabetes 2013 [36]	South India	1545	1304							-	-	-	-	-	-	-	-
	North India	5193	4493							-	-	-	-	-	-	-	-
Uma Jyothi PLoS One 2013 [37]	South India	758	621	52.5 (9.08)	52.2 (7.55)	6.41 (1.8)	-	27.07 (4.63)	24.72 (4.62)	-	-	-	-	1.69 (0.43)	0.94 (0.03)	58.4	63.6
Tariq Mol Vis 2013 [38]	Pakistan	373	200	-	-	-	-	-	-	-	-	-	-			48.5	50.0

1. Tai ES, Corella D, Deurenberg-Yap M, et al. (2004) Differential effects of the C1431T and Pro12Ala PPARgamma gene variants on plasma lipids and diabetes risk in an Asian population. J Lipid Res 45:674–85. doi: 10.1194/jlr.M300363-JLR200

2. Radha V, Vimaleswaran KS, Babu HNS, et al. (2006) Role of genetic polymorphism peroxisome proliferator-activated receptor-gamma2 Pro12Ala on ethnic susceptibility to diabetes in South-Asian and Caucasian subjects: Evidence for heterogeneity. Diabetes Care 29:1046–51. doi: 10.2337/diacare.2951046

3. Humphries SE, Gable D, Cooper JA, et al. (2006) Common variants in the TCF7L2 gene and predisposition to type 2 diabetes in UK European Whites, Indian Asians and Afro-Caribbean men and women. J Mol Med (Berl) 84:1005–14.

4. Chandak GR, Janipalli CS, Bhaskar S, et al. (2007) Common variants in the TCF7L2 gene are strongly associated with type 2 diabetes mellitus in the Indian population. Diabetologia 50:63–7. doi: 10.1007/s00125-006-0502-2

5. Bodhini D, Radha V, Dhar M, et al. (2007) The rs12255372(G/T) and rs7903146(C/T) polymorphisms of the TCF7L2 gene are associated with type 2 diabetes mellitus in Asian Indians. Metabolism 56:1174–8. doi: 10.1016/j.metabol.2007.04.012

6. Sanghera DK, Ortega L, Han S, et al. (2008) Impact of nine common type 2 diabetes risk polymorphisms in Asian Indian Sikhs: PPARG2 (Pro12Ala), IGF2BP2, TCF7L2 and FTO variants confer a significant risk. BMC Med Genet 9:59. doi: 10.1186/1471-2350-9-59

7. Sanghera DK, Nath SK, Ortega L, et al. (2008) TCF7L2 polymorphisms are associated with type 2 diabetes in Khatri Sikhs from North India: genetic variation affects lipid levels. Ann Hum Genet 72:499–509. doi: 10.1111/j.1469-1809.2008.00443.x

8. Rees SD, Bellary S, Britten AC, et al. (2008) Common variants of the TCF7L2 gene are associated with increased risk of type 2 diabetes mellitus in a UK-resident South Asian population. BMC Med Genet 9:8. doi: 10.1186/1471-2350-9-8

9. Sanghera DK, Been L, Ortega L, et al. (2009) Testing the association of novel meta-analysis-derived diabetes risk genes with type II diabetes and related metabolic traits in Asian Indian Sikhs. J Hum Genet 54:162–8. doi: 10.1038/jhg.2009.7

10. Yajnik CS, Janipalli CS, Bhaskar S, et al. (2009) FTO gene variants are strongly associated with type 2 diabetes in South Asian Indians. Diabetologia 52:247–52. doi: 10.1007/s00125-008-1186-6

11. Haseeb A, Iliyas M, Chakrabarti S, et al. (2009) Single-nucleotide polymorphisms in peroxisome proliferator-activated receptor gamma and their association with plasma levels of resistin and the metabolic syndrome in a South Indian population. J Biosci 34:405–14.

12. Chauhan G, Spurgeon CJ, Tabassum R, et al. (2010) Impact of common variants of PPARG, KCNJ11, TCF7L2, SLC30A8, HHEX, CDKN2A, IGF2BP2, and CDKAL1 on the risk of type 2 diabetes in 5,164 Indians. Diabetes 59:2068–74. doi: 10.2337/db09-1386

13. Gupta V, Khadgawat R, Ng HKT, et al. (2010) A validation study of type 2 diabetes-related variants of the TCF7L2, HHEX, KCNJ11, and ADIPOQ genes in one endogamous ethnic group of north India. Ann Hum Genet 74:361–8. doi: 10.1111/j.1469-1809.2010.00580.x

14. Chidambaram M, Radha V, Mohan V (2010) Replication of recently described type 2 diabetes gene variants in a South Indian population. Metabolism 59:1760–6. doi: 10.1016/j.metabol.2010.04.024

15. Mukhopadhyaya PN, Acharya A, Chavan Y, et al. (2010) Metagenomic study of single-nucleotide polymorphism within candidate genes associated with type 2 diabetes in an Indian population. Genet Mol Res 9:2060–8. doi: 10.4238/vol9-4gmr883

16. Sanghera DK, Demirci FY, Been L, et al. (2010) PPARG and ADIPOQ gene polymorphisms increase type 2 diabetes mellitus risk in Asian Indian Sikhs: Pro12Ala still remains as the strongest predictor. Metabolism 59:492–501. doi: 10.1016/j.metabol.2009.07.043

17. Vimaleswaran KS, Radha V, Jayapriya MG, et al. (2010) Evidence for an association with type 2 diabetes mellitus at the PPARG locus in a South Indian population. Metabolism 59:457–62. doi: 10.1016/j.metabol.2009.07.034

18. Tan JT, Ng DPK, Nurbaya S, et al. (2010) Polymorphisms identified through genome-wide association studies and their associations with type 2 diabetes in Chinese, Malays, and Asian-Indians in Singapore. J Clin Endocrinol Metab 95:390–7. doi: 10.1210/jc.2009-0688

19. Rees SD, Islam M, Hydrie MZI, et al. (2011) An FTO variant is associated with Type 2 diabetes in South Asian populations after accounting for body mass index and waist circumference. Diabet Med 28:673–80. doi: 10.1111/j.1464-5491.2011.03257.x

20. Rees SD, Hydrie MZI, O'Hare JP, et al. (2011) Effects of 16 genetic variants on fasting glucose and type 2 diabetes in South Asians: ADCY5 and GLIS3 variants may predispose to type 2 diabetes. PLoS One 6:e24710. doi: 10.1371/journal.pone.0024710

21. Chavali S, Mahajan A, Tabassum R, et al. (2011) Association of variants in genes involved in pancreatic β-cell development and function with type 2 diabetes in North Indians. J Hum Genet 56:695–700. doi: 10.1038/jhg.2011.83

22. Boodram LG, Miyake K, Hayes MG, et al. (2011) Association of the KCNJ11 variant E23K with type 2 diabetes in Indo-Trinidadians. West Indian Med J 60:604–7.

23. Rees SD, Hydrie MZI, Shera AS, et al. (2011) Replication of 13 genome-wide association (GWA)-validated risk variants for type 2 diabetes in Pakistani populations. Diabetologia 54:1368–74. doi: 10.1007/s00125-011-2063-2

24. Sim X, Ong RT-H, Suo C, et al. (2011) Transferability of type 2 diabetes implicated loci in multi-ethnic cohorts from Southeast Asia. PLoS Genet 7:e1001363. doi: 10.1371/journal.pgen.1001363

25. Chauhan G, Tabassum R, Mahajan A, et al. (2011) Common variants of FTO and the risk of obesity and type 2 diabetes in Indians. J Hum Genet 56:720–6. doi: 10.1038/jhg.2011.87

26. Anuradha S, Radha V, Mohan V (2011) Association of novel variants in the hepatocyte nuclear factor 4A gene with maturity onset diabetes of the young and early onset type 2 diabetes. Clin Genet 80:541–9. doi: 10.1111/j.1399-0004.2010.01577.x

27. Kooner JS, Saleheen D, Sim X, et al. (2011) Genome-wide association study in individuals of South Asian ancestry identifies six new type 2 diabetes susceptibility loci. Nat Genet 43:984–9. doi: 10.1038/ng.921

28. Been LF, Ralhan S, Wander GS, et al. (2011) Variants in KCNQ1 increase type II diabetes susceptibility in South Asians: a study of 3,310 subjects from India and the US. BMC Med Genet 12:18. doi: 10.1186/1471-2350-12-18

29. Ramya K, Radha V, Ghosh S, et al. (2011) Genetic variations in the FTO gene are associated with type 2 diabetes and obesity in south Indians (CURES-79). Diabetes Technol Ther 13:33–42. doi: 10.1089/dia.2010.0071

30. Janipalli CS, Kumar MVK, Vinay DG, et al. (2012) Analysis of 32 common susceptibility genetic variants and their combined effect in predicting risk of Type 2 diabetes and related traits in Indians. Diabet Med 29:121–7. doi: 10.1111/j.1464-5491.2011.03438.x

31. Been LF, Hatfield JL, Shankar A, et al. (2012) A low frequency variant within the GWAS locus of MTNR1B affects fasting glucose concentrations: genetic risk is modulated by obesity. Nutr Metab Cardiovasc Dis 22:944–51. doi: 10.1016/j.numecd.2011.01.006

32. Raza ST, Abbas S, Ahmed F, et al. (2012) Association of MTHFR and PPARγ2 gene polymorphisms in relation to type 2 diabetes mellitus cases among north Indian population. Gene 511:375–9. doi: 10.1016/j.gene.2012.09.072

33. Anand SS, Meyre D, Pare G, et al. (2013) Genetic information and the prediction of incident type 2 diabetes in a high-risk multiethnic population: the EpiDREAM genetic study. Diabetes Care 36:2836–42. doi: 10.2337/dc12-2553

34. Ali S, Chopra R, Manvati S, et al. (2013) Replication of type 2 diabetes candidate genes variations in three geographically unrelated Indian population groups. PLoS One 8:e58881. doi: 10.1371/journal.pone.0058881

35. Saxena R, Saleheen D, Been LF, et al. (2013) Genome-wide association study identifies a novel locus contributing to type 2 diabetes susceptibility in Sikhs of Punjabi origin from India. Diabetes 62:1746–55. doi: 10.2337/db12-1077

36. Tabassum R, Chauhan G, Dwivedi OP, et al. (2013) Genome-wide association study for type 2 diabetes in Indians identifies a new susceptibility locus at 2q21. Diabetes 62:977–86. doi: 10.2337/db12-0406

37. Uma Jyothi K, Jayaraj M, Subburaj KS, et al. (2013) Association of TCF7L2 gene polymorphisms with T2DM in the population of Hyderabad, India. PLoS One 8:e60212. doi: 10.1371/journal.pone.0060212

38. Tariq K, Malik SB, Ali SHB, et al. (2013) Association of Pro12Ala polymorphism in peroxisome proliferator activated receptor gamma with proliferative diabetic retinopathy. Mol Vis 19:710–7.
