# Supplementary material for: Does genetic heterogeneity account for the divergent risk of type 2 diabetes in South Asian and white European populations?
Source: Diabetologia. 2014 Aug 22;57(11):2270–81. doi: 10.1007/s00125-014-3354-1 (PMC4180911; doi:10.1007/s00125-014-3354-1)
Supplement: Supplementary file 4 — (PDF 113 kb) [file 125_2014_3354_MOESM4_ESM.pdf]

Electronic Supplementary Material Table 4 – Risk of bias assessment for studies included in the systematic review

**Reference**	**HWE tested in controls**	**Genotyping call rate > 95%**	**Source of cases**	**Source of controls**
Tai J Lipid Res 2004 [1]	Y	not reported; 20% re-genotyped with 100% concordance	general population	general population
Radha Diabetes Care 2006 [2]	Y	not reported	general population	general population
Humphries J Mol Med 2006 [3]	Y	not reported	WHSS: recruited from general practice clinics, PREDICT: recruited from the diabetes clinics, UCS: recruited from the diabetes clinics	WHSS: recruited from general practice clinics
Chandak Diabetologia 2007 [4]	Y	not reported; 15% were re-genotyped with 99.9% concordance	recruited from Diabetes Clinic of the King Edward Memorial Hospital and Research Centre in Pune	parents of children in the Pune Maternal Nutrition Study
Bodhini Clin Exp Met 2007 [5]	Y	not reported; 20% re-genotyped with 99% concordance	general population	general population
Sanghera BMC Med Genet 2008 [6]	Y	Y	endogamous Khatri Sikh population	endogamous Khatri Sikh population with no family history of type 2 diabetes; 262 were non-diabetic spouses
Sanghera Ann Hum Genet 2008 [7]	Y	Y	endogamous Khatri Sikh population	endogamous Khatri Sikh population with no family history of type 2 diabetes; 262 were non-diabetic spouses
Rees BMC Med Genet 2008 [8]	Y	Y	not specified	same geographical areas through community screening
Sanghera J Human Genet 2009 [9]	Y	Y	endogamous Khatri Sikh population	endogamous Khatri Sikh population with no family history of type 2 diabetes; 262 were non-diabetic spouses

Yajnik Diabetologia 2009 [10]	Y	not reported	recruited from Diabetes Clinic of the King Edward Memorial Hospital and Research Centre in Pune	parents of children in the Pune Maternal Nutrition Study from urban and rural regions, parents in Pune Children Study, and Coronary Risk of Insulin Sensitivity in Indian Subjects study
Haseeb J Biosci 2009 [11]	Y	not reported; subset re-genotyped	recruited from Mediciti hospital	recruited from Mediciti hospital
Chauhan Diabetes 2010 [12]	Y	>90%, 10% with concordance of 99.7%	Delhi: consecutively recruited from the Endocrinology clinic of All India Institute of Medical Sciences; Pune: general population in Pune and surrounding areas	Delhi: urban dwellers of Indo-European ethnicity with no family history of diabetes in first and/or second degree relatives; Pune: parents of children in the Pune Maternal Nutrition Study and Coronary Risk of Insulin Sensitivity in Indian Subjects study
Gupta Ann Hum Genet 2010 [13]	Y	not reported	Aggarwal population (60% had family history of type 2 diabetes)	same geographic region as cases
Chidambaram Metabolism 2010 [14]	Y	not reported; 20% re-genotyped with 99.6% concordance	general population	general population
Mukhopadhyaya Genet Mol Res 2010 [15]	Y	Y	pre-specified sub-population	same population as cases
Sanghera Metabolism 2010 [16]	Y	Y	endogamous Khatri Sikh population	endogamous Khatri Sikh population with no family history of type 2 diabetes; 262 were non-diabetic spouses
Vimaleswaran Met Clin Exp 2010 [17]	Y	not reported; 20% re-genotyped with 100% concordance	general population	general population
Tan J Clin End 2010 [18]	Y (rs2237892 and rs2237897 deviated from HWE $p<0.01$)	>90% call rate; 30 samples re-genotyped with 100% concordance	SDCS: recruited from Singapore National Healthcare Group Polyclinics; NHS98: general population	NHS98: general population

Rees Diabet Med 2011 [19]	Y	Y	Cobra: community sampling; UKADS: not specified; DGP: hospitals in Mirpur	Cobra: community sampling; UKADS: same geographic region; DGP: community screening
Rees PloS One 2011 [20]	Y	Y	UKADS: not specified; DGP: hospitals in Mirpur	UKADS: same geographic region; DGP: community screening
Chavali J Human Genet 2011 [21]	Y	Y	consecutively recruited from the Endocrinology clinic of All India Institute of Medical Sciences	urban dwellers of Indo-European ethnicity with no family history of diabetes in first and/or second degree relatives
Boodram West Indian Med J 2011 [22]	Y	not reported	recruited from diabetic outpatient clinics	recruited from Chest Clinic at Mt. Hope Hospital (Champs Fleurs, Trinidad and Tobago) and from San Fernando General Hospital
Rees Diabetologia 2011 [23]	Y	Y	UKADS: not specified; DGP: hospitals in Mirpur	UKADS: same geographic region; DGP: community screening
Sim PLoS Genet 2011 [24]	Y	Y	randomly sampled from general population	randomly sampled from general population
Chauhan J Hum Genet 2011 [25]	Y	>90%, 5% with concordance of 99.99%	consecutively recruited from the Endocrinology clinic of All India Institute of Medical Sciences	recruited from diabetes awareness camps
Anuradha Clin Genet 2011 [26]	Y	not reported; 20% re-genotyped with 99% concordance	general population	general population

Kooner Nat Genet 2011 [27]	Y	Y	LOLIPOP: recruited from lists of GPs in West London; PROMIS: out-patient departments; SINDI: general population; COBRA: general population; DGP: hospitals or Diabetes awareness camps; CURES: general population; Mauritius cohort: population based survey; RHS: population based survey used to participate in study; SDS: endogamous Khatri Sikh population; SCCS: recruited from hospitals and polyclinics	LOLIPOP: recruited from lists of GPs in West London; PROMIS: matched cases from visitors in out-patient; SINDI: general population; COBRA: general population; DGP: recruited from community screening camps; CURES: random sampling from general population; Mauritius cohort: population based survey; RHS: population based survey; SDS: endogamous Khatri Sikh population with no family history of type 2 diabetes, 262 were non-diabetic spouses; SCCS: general population
Been BMC Med Genet 2011 [28]	Y	Y	SDS: endogamous Khatri Sikh population; US cohort: not specified	SDS: endogamous Khatri Sikh population with no family history of type 2 diabetes, 262 were non-diabetic spouses; US cohort: public advertisement for free health screening
Ramya Diabetes Technol Ther 2011 [29]	Y	not reported; 20% re-genotyped with 99% concordance	general population	general population
Janipali Diabetic Med 2012 [30]	Y	Y	general population in Pune and surrounding areas	parents of children in the Pune Maternal Nutrition Study from urban and rural regions and Coronary Risk of Insulin Sensitivity in Indian Subjects study

Been Nutr Metab 2012 [31]	Y	Y	endogamous Khatri Sikh population	endogamous Khatri Sikh population with no family history of type 2 diabetes; 262 were non-diabetic spouses
Raza Gene 2012 [32]	not reported	not reported	recruited from a diabetic clinic	recruited from same diabetic clinic with no history of type 2 diabetes
Anand Diabetes Care 2013 [33]	Y	Y	Individuals at risk for dysglycemia recruited from 191 centres around the world via a variety of methods	Sample population as cases
Ali PLoS One 2013 [34]	Y	Y	-	-
Saxena Diabetes 2013 [35]	Y	Y	LOLIPOP: recruited from lists of GPs in West London; PROMIS: out-patient departments; SINDI: general population; DGP: hospitals or Diabetes awareness camps; CURES: general population; SDS: endogamous Khatri Sikh population; RACE: recruited from six hospital centres in Pakistan; UKADS: general population; SLDS: general population	LOLIPOP: recruited from lists of GPs in West London; PROMIS: matched cases from visitors in out-patient; SINDI: general population; DGP: recruited from community screening camps; CURES: random sampling from general population; SDS: endogamous Khatri Sikh population with no family history of type 2 diabetes, 262 were non-diabetic spouses RACE: recruited from six hospital centres in Pakistan; UKADS: general population (same geographical area); SLDS: general population
Tabassum Diabetes 2013 [36]	Y	Y	INDICO: consecutively recruited from the Endocrinology clinic of All India Institute of Medical Sciences; CURES: general population	INDICO: recruited from diabetes awareness camps; CURES: general population

Uma Jyothi PLoS One 2013 [37]	Y	Y	recruited from J.P. Endocrine center	community diabetic camp
Tariq Mol Vis 2013 [38]	Y	not reported; 10% replicated with 100% concordance	recruited from hospitals	not specified

1. Tai ES, Corella D, Deurenberg-Yap M, et al. (2004) Differential effects of the C1431T and Pro12Ala PPARgamma gene variants on plasma lipids and diabetes risk in an Asian population. J Lipid Res 45:674–85.

2. Radha V, Vimaleswaran KS, Babu HNS, et al. (2006) Role of genetic polymorphism peroxisome proliferator-activated receptor-gamma2 Pro12Ala on ethnic susceptibility to diabetes in South-Asian and Caucasian subjects: Evidence for heterogeneity. Diabetes Care 29:1046–51.

3. Humphries SE, Gable D, Cooper JA, et al. (2006) Common variants in the TCF7L2 gene and predisposition to type 2 diabetes in UK European Whites, Indian Asians and Afro-Caribbean men and women. J Mol Med (Berl) 84:1005–14.

4. Chandak GR, Janipalli CS, Bhaskar S, et al. (2007) Common variants in the TCF7L2 gene are strongly associated with type 2 diabetes mellitus in the Indian population. Diabetologia 50:63–7.

5. Bodhini D, Radha V, Dhar M, et al. (2007) The rs12255372(G/T) and rs7903146(C/T) polymorphisms of the TCF7L2 gene are associated with type 2 diabetes mellitus in Asian Indians. Metabolism 56:1174–8.

6. Sanghera DK, Ortega L, Han S, et al. (2008) Impact of nine common type 2 diabetes risk polymorphisms in Asian Indian Sikhs: PPARG2 (Pro12Ala), IGF2BP2, TCF7L2 and FTO variants confer a significant risk. BMC Med Genet 9:59.

7. Sanghera DK, Nath SK, Ortega L, et al. (2008) TCF7L2 polymorphisms are associated with type 2 diabetes in Khatri Sikhs from North India: genetic variation affects lipid levels. Ann Hum Genet 72:499–509.

8. Rees SD, Bellary S, Britten AC, et al. (2008) Common variants of the TCF7L2 gene are associated with increased risk of type 2 diabetes mellitus in a UK-resident South Asian population. BMC Med Genet 9:8.

9. Sanghera DK, Been L, Ortega L, et al. (2009) Testing the association of novel meta-analysis-derived diabetes risk genes with type II diabetes and related metabolic traits in Asian Indian Sikhs. J Hum Genet 54:162–8.

10. Yajnik CS, Janipalli CS, Bhaskar S, et al. (2009) FTO gene variants are strongly associated with type 2 diabetes in South Asian Indians. Diabetologia 52:247–52.

11. Haseeb A, Iliyas M, Chakrabarti S, et al. (2009) Single-nucleotide polymorphisms in peroxisome proliferator-activated receptor gamma and their association with plasma levels of resistin and the metabolic syndrome in a South Indian population. J Biosci 34:405–14.

12. Chauhan G, Spurgeon CJ, Tabassum R, et al. (2010) Impact of common variants of PPARG, KCNJ11, TCF7L2, SLC30A8, HHEX, CDKN2A, IGF2BP2, and CDKAL1 on the risk of type 2 diabetes in 5,164 Indians. Diabetes 59:2068–74.

13. Gupta V, Khadgawat R, Ng HKT, et al. (2010) A validation study of type 2 diabetes-related variants of the TCF7L2, HHEX, KCNJ11, and ADIPOQ genes in one endogamous ethnic group of north India. Ann Hum Genet 74:361–8.

14. Chidambaram M, Radha V, Mohan V (2010) Replication of recently described type 2 diabetes gene variants in a South Indian population. Metabolism 59:1760–6.

15. Mukhopadhyaya PN, Acharya A, Chavan Y, et al. (2010) Metagenomic study of single-nucleotide polymorphism within candidate genes associated with type 2 diabetes in an Indian population. Genet Mol Res 9:2060–8.

16. Sanghera DK, Demirci FY, Been L, et al. (2010) PPARG and ADIPOQ gene polymorphisms increase type 2 diabetes mellitus risk in Asian Indian Sikhs: Pro12Ala still remains as the strongest predictor. Metabolism 59:492–501.

17. Vimaleswaran KS, Radha V, Jayapriya MG, et al. (2010) Evidence for an association with type 2 diabetes mellitus at the PPARG locus in a South Indian population. Metabolism 59:457–62.

18. Tan JT, Ng DPK, Nurbaya S, et al. (2010) Polymorphisms identified through genome-wide association studies and their associations with type 2 diabetes in Chinese, Malays, and Asian-Indians in Singapore. J Clin Endocrinol Metab 95:390–7.

19. Rees SD, Islam M, Hydrie MZI, et al. (2011) An FTO variant is associated with Type 2 diabetes in South Asian populations after accounting for body mass index and waist circumference. Diabet Med 28:673–80.

20. Rees SD, Hydrie MZI, O'Hare JP, et al. (2011) Effects of 16 genetic variants on fasting glucose and type 2 diabetes in South Asians: ADCY5 and GLIS3 variants may predispose to type 2 diabetes. PLoS One 6:e24710.

21. Chavali S, Mahajan A, Tabassum R, et al. (2011) Association of variants in genes involved in pancreatic β-cell development and function with type 2 diabetes in North Indians. J Hum Genet 56:695–700.

22. Boodram LG, Miyake K, Hayes MG, et al. (2011) Association of the KCNJ11 variant E23K with type 2 diabetes in Indo-Trinidadians. West Indian Med J 60:604–7.

23. Rees SD, Hydrie MZI, Shera AS, et al. (2011) Replication of 13 genome-wide association (GWA)-validated risk variants for type 2 diabetes in Pakistani populations. Diabetologia 54:1368–74.

24. Sim X, Ong RT-H, Suo C, et al. (2011) Transferability of type 2 diabetes implicated loci in multi-ethnic cohorts from Southeast Asia. PLoS Genet 7:e1001363.

25. Chauhan G, Tabassum R, Mahajan A, et al. (2011) Common variants of FTO and the risk of obesity and type 2 diabetes in Indians. J Hum Genet 56:720–6.

26. Anuradha S, Radha V, Mohan V (2011) Association of novel variants in the hepatocyte nuclear factor 4A gene with maturity onset diabetes of the young and early onset type 2 diabetes. Clin Genet 80:541–9.

27. Kooner JS, Saleheen D, Sim X, et al. (2011) Genome-wide association study in individuals of South Asian ancestry identifies six new type 2 diabetes susceptibility loci. Nat Genet 43:984–9.

28. Been LF, Ralhan S, Wander GS, et al. (2011) Variants in KCNQ1 increase type II diabetes susceptibility in South Asians: a study of 3,310 subjects from India and the US. BMC Med Genet 12:18.

29. Ramya K, Radha V, Ghosh S, et al. (2011) Genetic variations in the FTO gene are associated with type 2 diabetes and obesity in south Indians (CURES-79). Diabetes Technol Ther 13:33–42.

30. Janipalli CS, Kumar MVK, Vinay DG, et al. (2012) Analysis of 32 common susceptibility genetic variants and their combined effect in predicting risk of Type 2 diabetes and related traits in Indians. Diabet Med 29:121–7.

31. Been LF, Hatfield JL, Shankar A, et al. (2012) A low frequency variant within the GWAS locus of MTNR1B affects fasting glucose concentrations: genetic risk is modulated by obesity. Nutr Metab Cardiovasc Dis 22:944–51.

32. Raza ST, Abbas S, Ahmed F, et al. (2012) Association of MTHFR and PPARγ2 gene polymorphisms in relation to type 2 diabetes mellitus cases among north Indian population. Gene 511:375–9.

33. Anand SS, Meyre D, Pare G, et al. (2013) Genetic information and the prediction of incident type 2 diabetes in a high-risk multiethnic population: the EpiDREAM genetic study. Diabetes Care 36:2836–42.

34. Ali S, Chopra R, Manvati S, et al. (2013) Replication of type 2 diabetes candidate genes variations in three geographically unrelated Indian population groups. PLoS One 8:e58881.

35. Saxena R, Saleheen D, Been LF, et al. (2013) Genome-wide association study identifies a novel locus contributing to type 2 diabetes susceptibility in Sikhs of Punjabi origin from India. Diabetes 62:1746–55.

36. Tabassum R, Chauhan G, Dwivedi OP, et al. (2013) Genome-wide association study for type 2 diabetes in Indians identifies a new susceptibility locus at 2q21. Diabetes 62:977–86.

37. Uma Jyothi K, Jayaraj M, Subburaj KS, et al. (2013) Association of TCF7L2 gene polymorphisms with T2DM in the population of Hyderabad, India. PLoS One 8:e60212.

38. Tariq K, Malik SB, Ali SHB, et al. (2013) Association of Pro12Ala polymorphism in peroxisome proliferator activated receptor gamma with proliferative diabetic retinopathy. Mol Vis 19:710–7.
